# Zooplankton biogeographic boundaries in the California Current System as determined from metabarcoding

**DOI:** 10.1371/journal.pone.0235159

**Published:** 2020-06-25

**Authors:** Kathleen J. Pitz, Jinchen Guo, Shannon B. Johnson, Tracy L. Campbell, Haibin Zhang, Robert C. Vrijenhoek, Francisco P. Chavez, Jonathan Geller

**Affiliations:** 1 Monterey Bay Aquarium Research Institute, Moss Landing, California, United States of America; 2 Moss Landing Marine Laboratories, Moss Landing, California, United States of America; University of Central Florida, UNITED STATES

## Abstract

Within the southern California Current ecosystem there are two well-documented breaks in marine community structure at Point Conception and Punta Eugenia. We explored the presence of similar breaks in a diverse zooplankton community through metabarcoding of mixed net tow tissue samples collected during an expedition from Monterey to Baja California in February of 2012. We recovered a high diversity of species as well as patterns of species presence that align with their previously documented ranges in this region. We found a clear break at Punta Eugenia in overall zooplankton community structure, while Point Conception was weakly linked to changes in community structure. We analyzed this dataset through two parallel bioinformatic pipelines to examine the robustness of these results. Our overall conclusions were consistent across both pipelines, however there were differences in species detection. This study illustrates the utility of metabarcoding analysis on mixed tissue samples for recovering known patterns of diversity, as well as allowing elucidation of broad patterns of community differentiation across many groups of organisms.

## Introduction

Ecologists have long been fascinated with biogeographic boundaries that separate regions of strikingly different biological communities. Along the southwestern coast of North America there are two well described biogeographic boundaries in marine community structure: the first at Point Conception in southern California and the second at Punta Eugenia in Baja California. These headlands separate different physical oceanographic regimes and are the location of many species range endpoints as well as documented barriers to gene flow [1–6]. Biological diversity across these breaks has been extensively studied, however, individual studies relying on traditional morphological methods are typically focused on select few taxa, aligned with researchers’ taxonomic expertise. Studies that incorporate information from multiple sources show these breaks can have varying influence on different taxa [7] and may represent ‘transitional zones’ rather than hard breaks [8]. Here we use genetic markers to target a broad range of nearshore zooplankton species to determine the degree to which Point Conception and Punta Eugenia result in hard breaks in zooplankton community structure or if communities exist in a continium from north to south latitudes.

**Fig 1:**
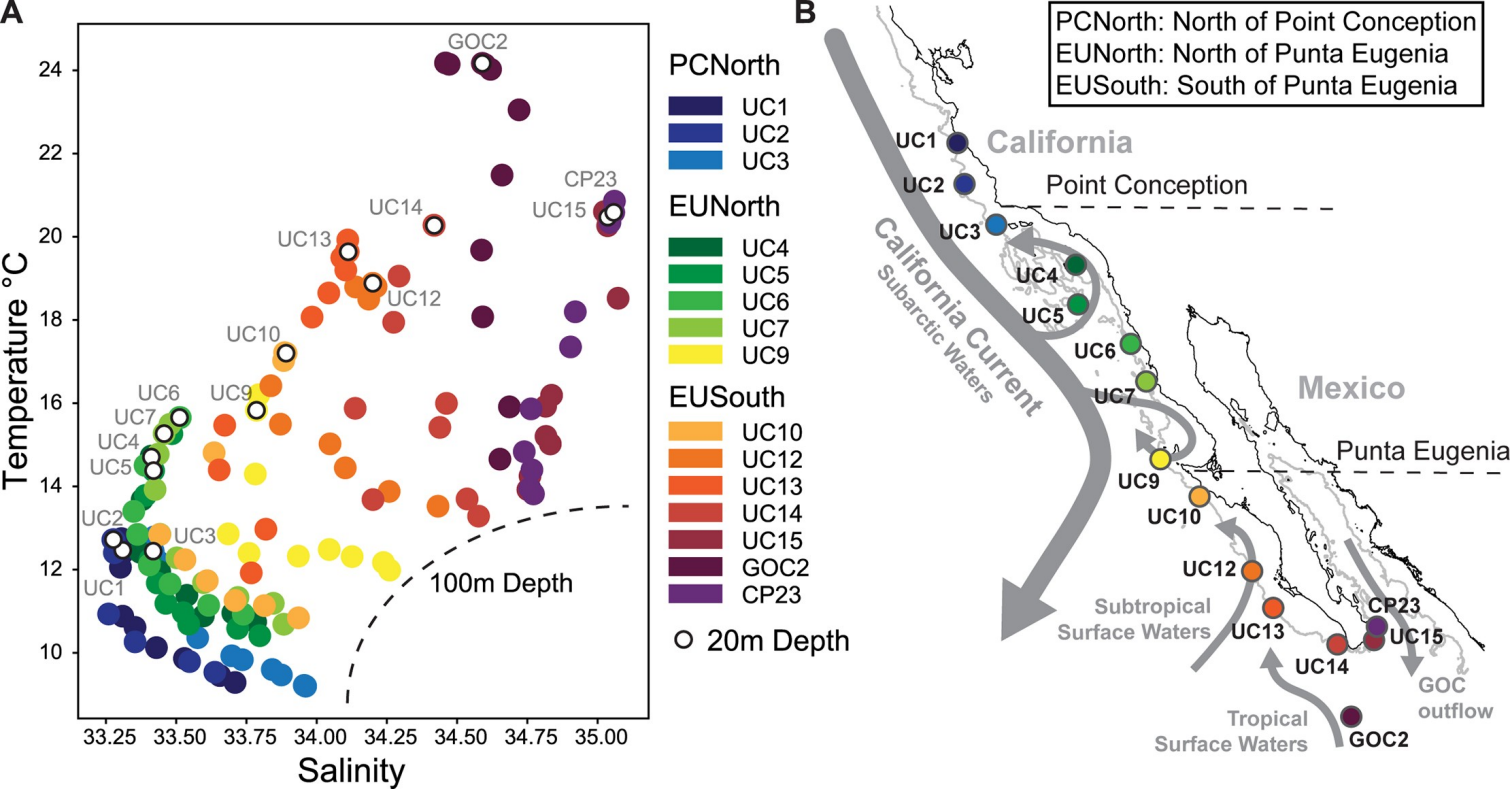
Environmental variability and sampling locations along cruise track. (A) Temperature versus salinity at each station at depths 0-100m showing 10m increments. Values at 20m are highlighted. (B) Location of each sampling station alongside 1000m bathymetric contour and an overview of the dominant water masses in the region. The California Current is shown as it runs offshore of the western coast of North America before diverging at Punta Eugenia and eventually feeding into the North Equatorial Current as part of the North Pacific Subtropical Gyre circulation.

Changing physical conditions at these breaks or zones are in part due to dynamics associated with the California Current (CC), which runs southward off the western coast of North America from British Columbia to Baja California (Fig 1). North of Point Conception a cooler ‘Oregonian’ regime dominates and much of the coast experiences strong winds and upwelling through spring and summer. At Point Conception, the CC is diverted offshore and a segment flows northward to seasonally form the Southern California Eddy within the Southern California Bight. Within the Southern California Bight winds are weaker and upwelling mainly occurs seasonally in winter and early spring. Further south, the CC runs again in closer proximity to shore before diverging entirely offshore at Punta Eugenia on the Baja California Peninsula. South of Punta Eugenia there is a stronger influence of subtropical and equatorial water masses as well as outflow from the Gulf of California (Fig 1)[9,10]. Both Pt. Conception and P. Eugenia are also the sites of large persistent eddies which can function to isolate zooplankton populations [11].

Zooplankton form a key link between trophic levels: their community structure and diversity affect both the primary producers they prey on and the higher trophic levels that consume them. Their community composition is also highly responsive to environmental variation, such as changes in currents and upwelling caused by capes [12], interannual changes in ocean conditions [13,14] and climate change [15–17].

Here we describe marine zooplankton community composition through metabarcoding of 15 mixed tissue samples collected in 100-meter depth, oblique net-tows, along the 1000-meter isobath of the west coast of the US and Mexico from February 4^th^-12^th^ 2012. We investigated how the marine zooplankton community varied along this latitudinal gradient involving significant changes in environmental parameters such as temperature and salinity and across two biogeographic breaks at Point Conception and Punta Eugenia. The results are compared to known biological patterns of this area. Our genetic analyses had the capability to detect and discriminate between organisms beyond what can be done with traditional morphological approaches, such as early life stages or morphologically similar species, which further enhanced this dataset. Genetic analyses were conducted using two different genetic markers, the small subunit ribosomal RNA (*18S* rRNA) gene and the mitochondrial cytochrome c oxidase subunit I (*COI*) gene, which were both analyzed through two parallel bioinformatic pipelines in order to examine the robustness of our results.

## Results

### Environmental variability

Sampling locations spanned large latitudinal variability in oceanographic conditions and crossed two documented biogeographic breaks in marine community structure (Fig 1, Table 1). North and south of Point Conception, from stations UC1 to UC9, conditions reflected the low temperature, low salinity subarctic waters that form the California Current. South of Punta Eugenia, from stations UC10 to CP23, we saw a transition to a greater influence of high salinity Gulf of California water (end members at CP23, UC15), subtropical surface water and high temperature tropical surface water (end member at GOC2) (Fig 1)[10,18]. The greatest range of temperature and salinity values occurred south of Punta Eugenia; values north and south of Point Conception were more similar (Fig 1).

**Table 1:** Sampling station locations and times.

Station	Region	Local Time PST (M/D/Y h:mm)	Time of Day	Minimum Time between sampling and sunrise/sunset (h:mm)	Latitude	Longitude
UC1	PCNorth	2/4/12 23:40	night	5:03	35.9998	-121.7698
UC2		2/5/12 8:40	sunrise	0:38	34.9998	-121.5600
UC3		2/5/12 20:00	sunset	1:23	34.0000	-120.6100
UC4	EUNorth	2/6/12 13:00	day	5:15	33.0000	-118.2300
UC5		2/6/12 23:59	night	5:28	32.0000	-118.1667
UC6		2/7/12 16:00	day	2:27	30.9998	-116.5598
UC7		2/8/12 0:30	night	6:03	30.0263	-116.1250
UC9		2/8/12 18:30	sunset	0:02	27.9933	-115.7003
UC10	EUSouth	2/9/12 6:30	sunrise	0:49	27.0043	-114.5317
UC12		2/10/12 4:00	night	3:10	24.9998	-112.9597
UC13		2/10/12 13:30	day	4:51	24.0003	-112.3285
UC14		2/11/12 4:00	night	2:57	23.0005	-110.4102
GOC2		2/11/12 0:30	night	6:14	20.9960	-109.9970
UC15		2/11/12 15:30	day	2:41	23.1195	-109.3158
CP23		2/12/12 22:05	night	3:54	23.4877	-109.2293

Sampling station locations and times (PST) and classification of samples as taken during night, day, sunset, or sunrise.

### Bioinformatic pipeline and marker gene comparison

**Fig 2:**
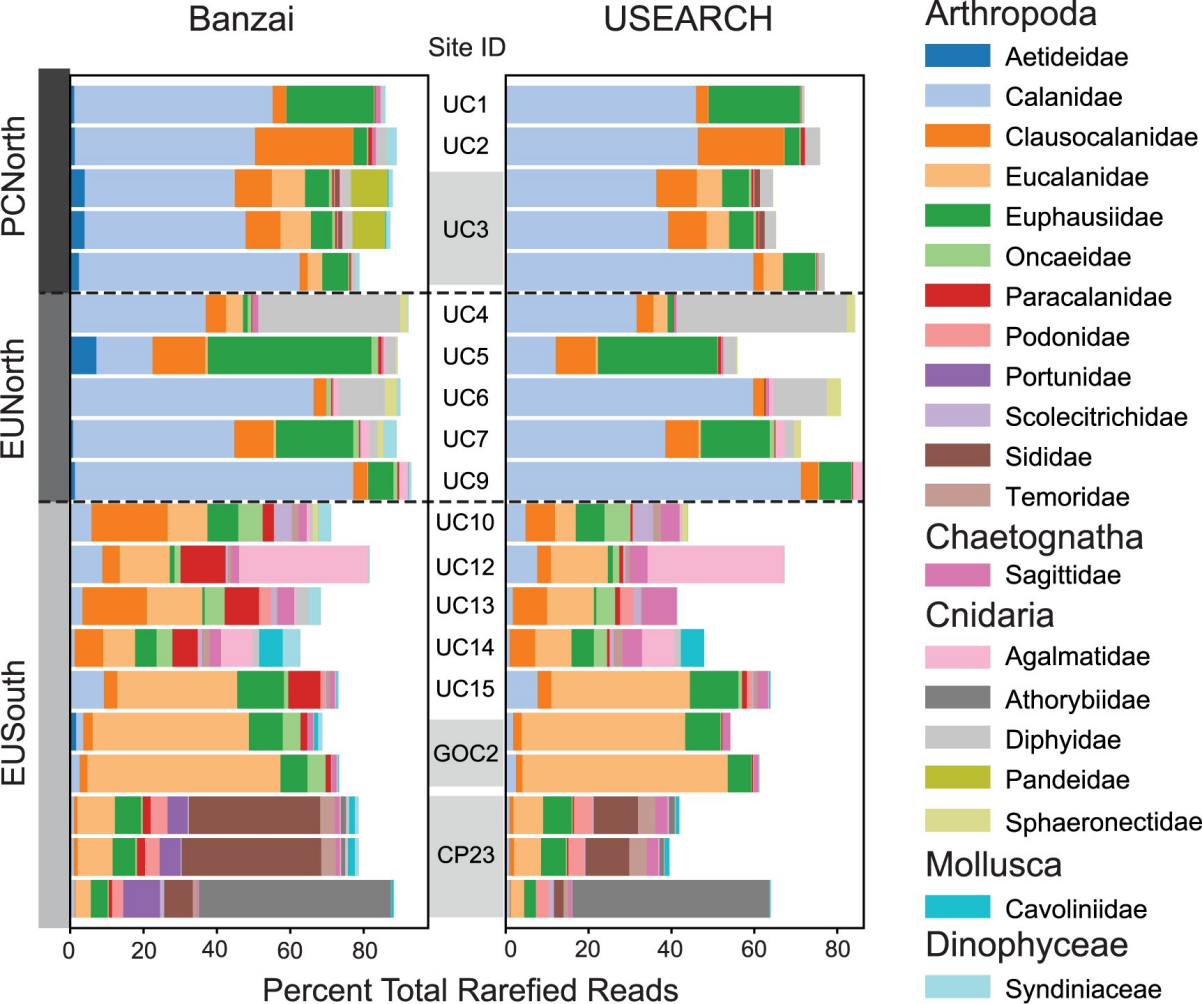
Most abundant families across Banzai and USEARCH pipelines.

A total of 20 environmental samples and one no-template control were sequenced resulting in 7,630,507 *COI* and 4,385,514 *18S* paired-end reads. These data were processed through two parallel bioinformatic pipelines, Banzai and USEARCH (described below in Materials and methods), and products from both pipelines were run through all following analyses. Both bioinformatic pipelines resulted in similar patterns of community composition although there were significant differences (Fig 2). Over a total of 20 environmental samples from 15 stations, we detected a large diversity of organisms dominated by arthropods, cnidarians, and molluscs. Notably one OTU was annotated to *Balaenoptera musculus* (Blue Whale), which also indicates the presence of sequences from environmental DNA (eDNA) within this dataset. The Banzai pipeline resulted in an order of magnitude greater number of OTUs than the USEARCH pipeline: 21,402 OTUs and 44,837 OTUs while the USEARCH pipeline resulted in 1,596 OTUs and 342 OTUs for *COI* and *18S* respectively. The major cause of this disparity was a difference in clustering method, such that a majority of Banzai OTUs clusters within USEARCH OTUs. However, sequence similarity between the OTUs recovered by both the Banzai and USEARCH pipelines was high. When blasted to the complete set of Banzai OTUs, 100% of USEARCH *COI* OTUs and 100% of USEARCH *18S* OTUs had a hit to a Banzai OTU with ≥95% identity and across ≥95% of USEARCH length. Within the Banzai pipeline results, 80.4% of *COI* OTUs and 97.2% of *18S* OTUs were assigned to a USEARCH OTU with ≥95% identity and across ≥95% of Banzai length. The majority of Banzai *COI* and *18S* OTUs that could not be assigned to USEARCH OTUs were annotated within the phylum Arthropoda and to classes Hexanauplia or Malacostraca or were not annotated. The USEARCH pipeline was also more stringent in the percent similarity of BLAST hits used to annotate OTUs, resulting in a greater proportion of OTUs that were unannotated. The Banzai pipeline allowed for annotation at a higher taxonomic level (such as phylum, order, or family) when genus and species limits were not met. Patterns of dominating families were preserved across pipelines (Fig 2) but overall more families were annotated in the Banzai pipeline than the USEARCH pipeline (314:100 Banzai:USEARCH *COI*; 177:69 Banzai:USEARCH *18S*). Within both pipelines the largest proportion of reads was annotated to the phylum Arthropoda and the majority of these were to copepods and krill.

The twenty most abundant families by total percent rarefied reads for the COI marker across both pipelines (Banzai and USEARCH). Replicates for sites UC3, GOC2, and CP23 are highlighted in grey within the Site ID column.

*18S* and *COI* markers within this study gave similar information at higher taxonomic levels but differed in how specifically they allowed taxonomic annotation between groups. For example, within the Banzai pipeline *COI* provided annotations at an increased taxonomic resolution for Arthropods (133:43 species *COI*:*18S*) and *18S* provided increased resolution for Dinoflagellates (7:13 species *COI*:*18S*). A higher percent of *COI* OTUs overall were assigned to a lower taxonomic level than *18S*. Frequently this illustrated either potential gaps in the reference sequence database or groups for which this target sequence is not as taxonomically informative. For example, within the *18S* Banzai dataset there were many sequences assigned to the family *Euphausiidae* or the genus *Euphausia*, while for the same samples, *COI* was able to assign sequences to *Nematoscelis difficilis* (a species within that family) as well as several different species within the genus *Euphausia* at a similar total proportion in the overall community.

### Regional differences in zooplankton across biogeographic boundaries

**Fig 3:**
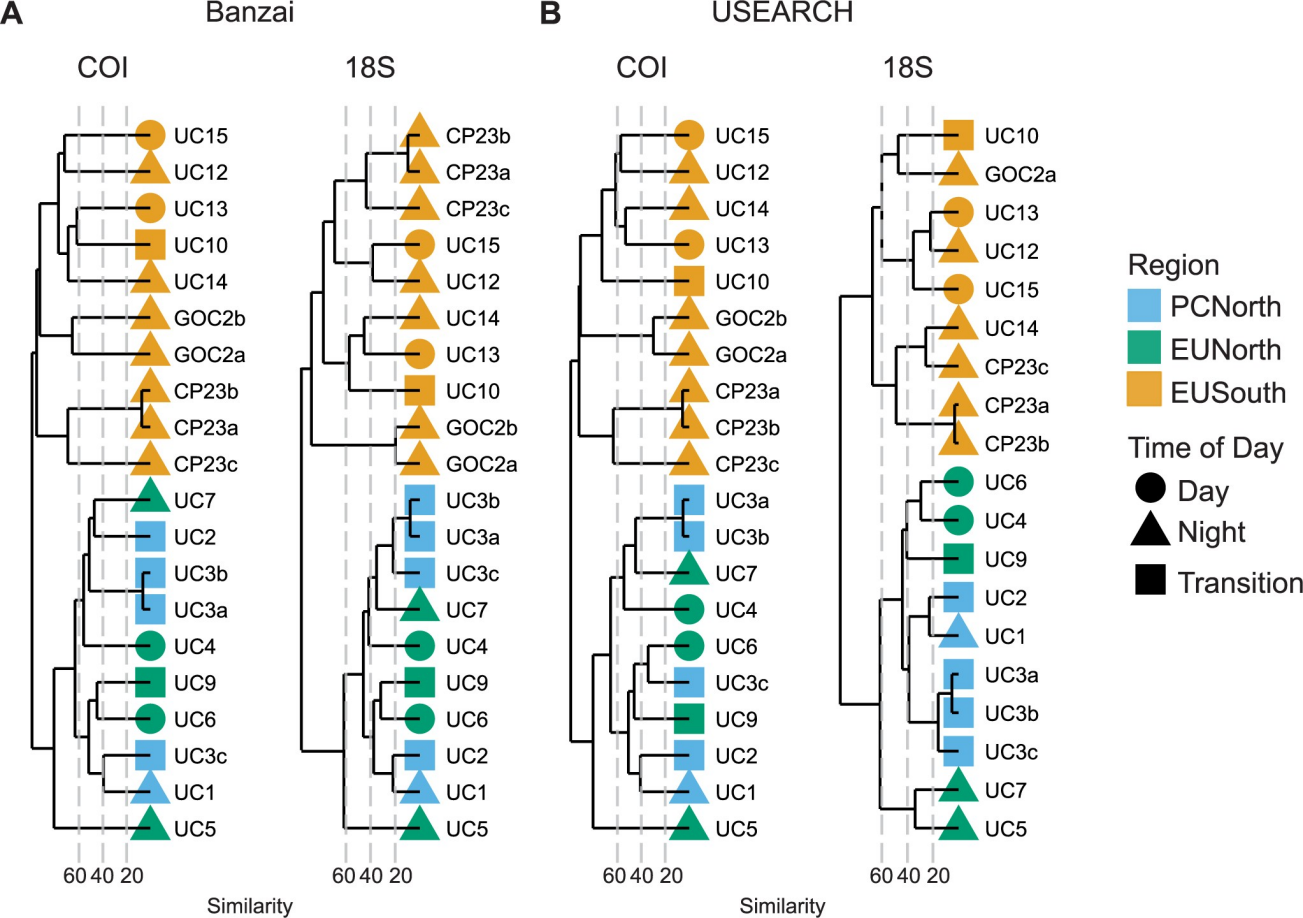
Hierarchical clustering of samples. Hierarchical clustering of samples by Bray-Curtis distance across both markers and pipelines.

**Fig 4:**
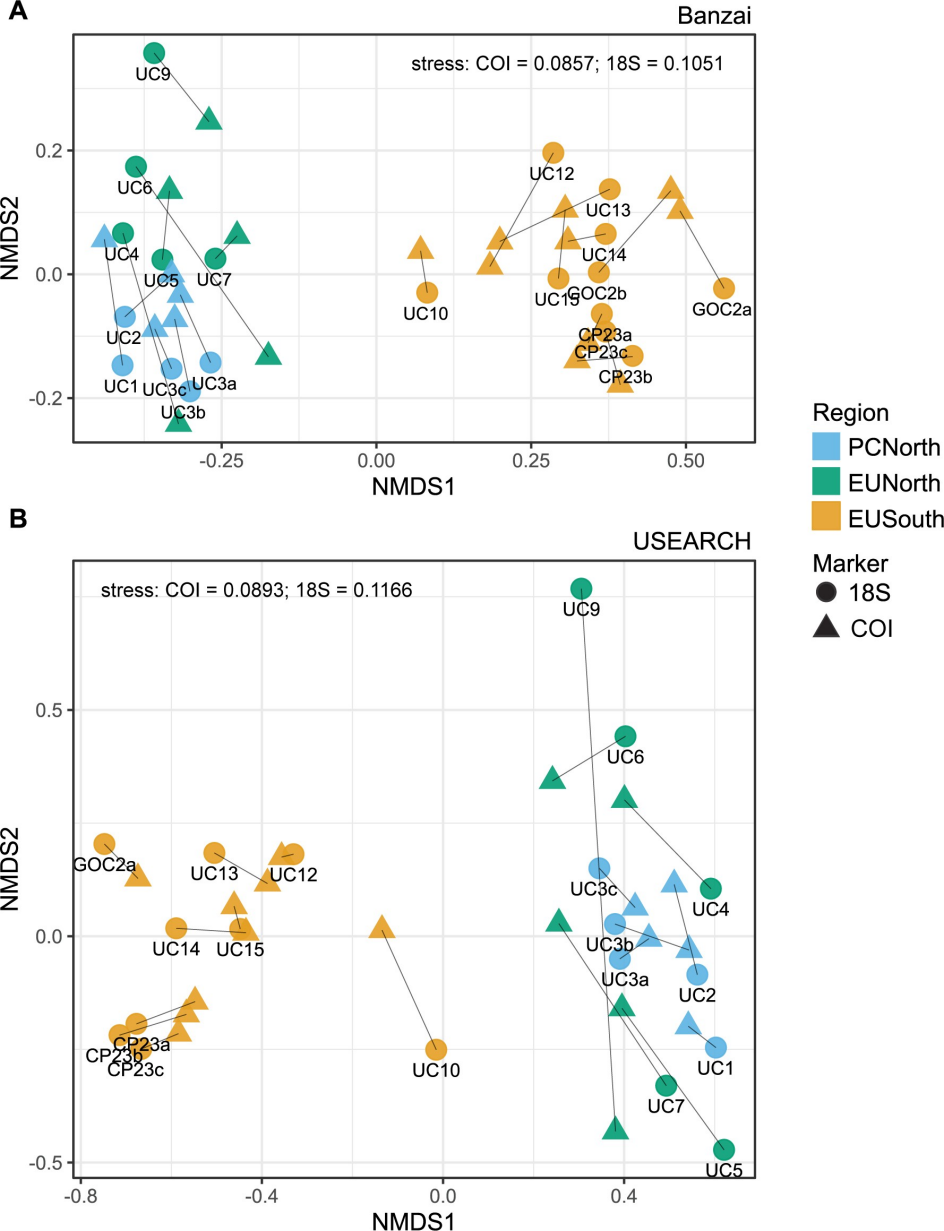
Procrustes analysis of NMDS plots. Comparison of clustering within NMDS plots by procrustes analysis with Bray-Curtis distance for (A) Banzai 18S and COI and (B) USEARCH 18S and COI.

**Fig 5:**
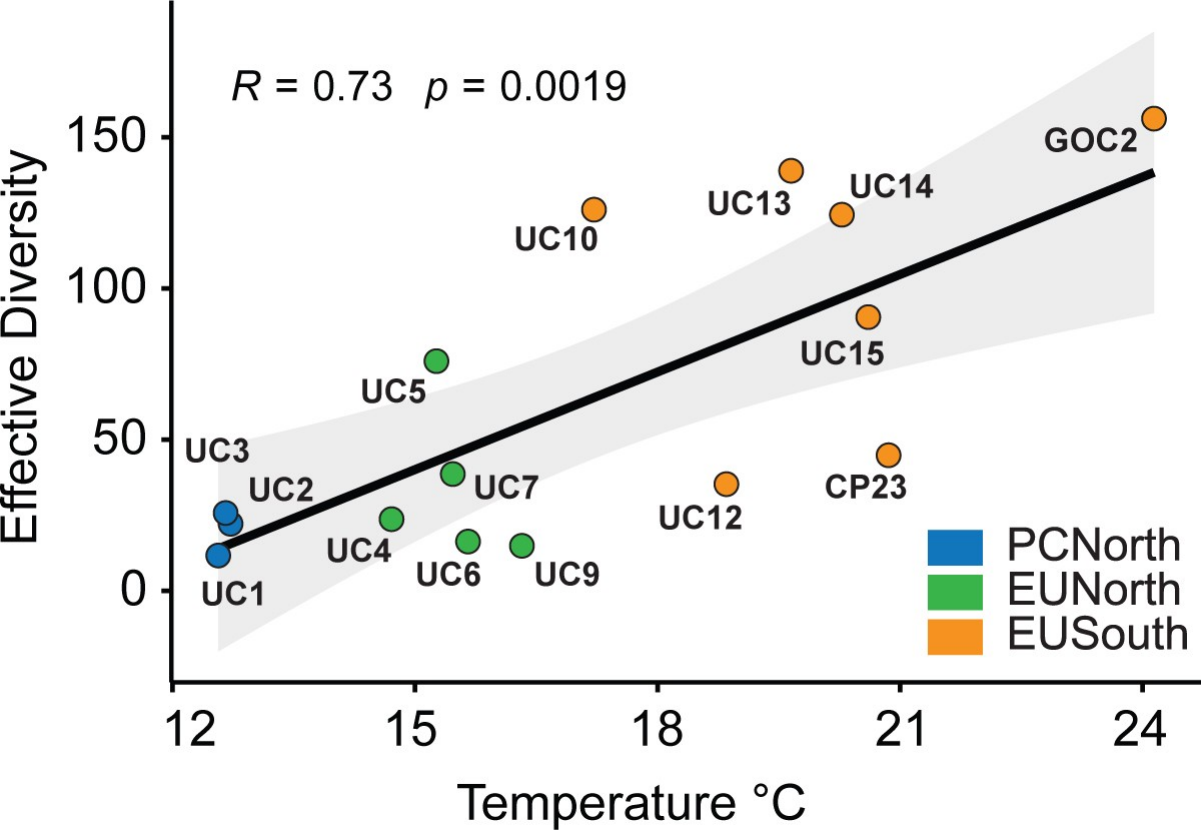
Effective diversity and temperature. Linear correlation between effective diversity and temperature across Banzai COI dataset. Mean diversity value of replicates was taken to represent sites UC3, CP23, and GOC2.

**Fig 6:**
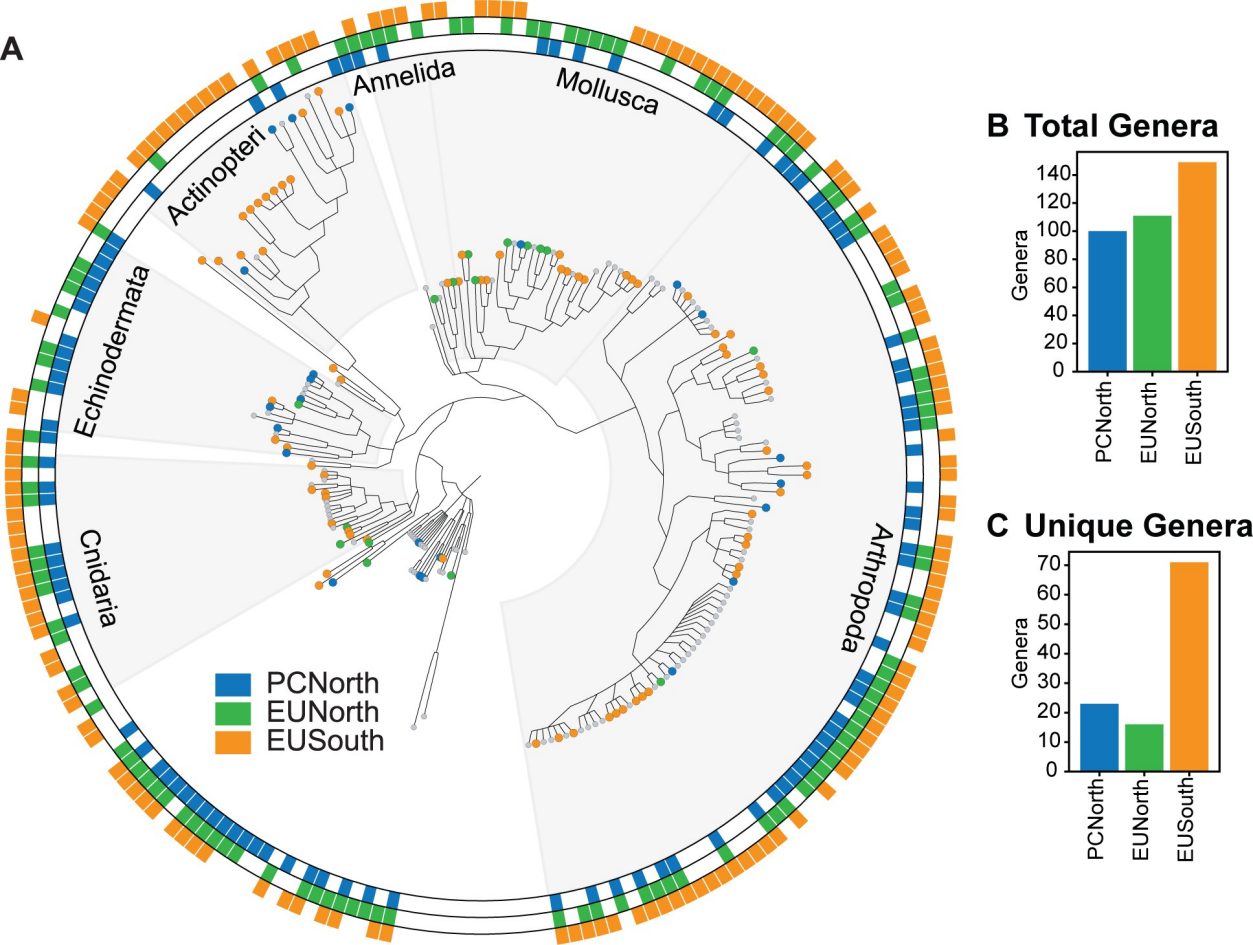
Detection of genera across regions. Detection of genus-level annotations within the COI Banzai dataset across the three regions: PCNorth (blue), EUNorth (green), and EUSouth (yellow). (A) Cladogram of genera detected. A colored bar within the ring indicates presence of OTUs annotated to that genus within the dataset. A blue, green, or yellow circle at the leaf of the tree indicates that genus was found only within that region. Highly abundant phyla are labeled. (B) Bar plot showing number of total genera found within each region. (C) Bar plot showing number of unique genera found within each region.

We found a significant difference in zooplankton community composition across Punta Eugenia but not Point Conception. Ordination and clustering analyses of samples run at the OTU level based on Bray-Curtis dissimilarity showed a break in community structure at Punta Eugenia with EUSouth samples consistently separating from EUNorth and PCNorth samples (Figs 3 and 4). There was less consistent clustering between samples taken north and south of Point Conception across both pipelines (Figs 3 and 4). Generally PCNorth samples clustered more closely together, but were encompassed by variability within EUNorth samples (Fig 4). Effective diversity values were positively correlated with temperature across this dataset (R = 0.73, p = 0.0019, Banzai COI Fig 5) and a higher number of total genera occurred in EUSouth samples than EUNorth or PCNorth samples (Fig 6B). Furthermore, EUSouth had greater numbers of genera unique to that region particularly among arthropods, molluscs (including 5 genera of pteropods), cnidarians, and fish (including 5 genera of myctophids) (Fig 6). An indicator of a different biogeographic region, the percent unique taxa for EUSouth was over 49% for Banzai COI data, much higher than EUNorth (16.7%) or PCNorth (19.4%) (S2 Table). Overall, as discussed below, at Punta Eugenia the community assemblage shifts to reflect the decreased influence of the California Current and the increased influence of equatorial, subtropical, and Gulf of California waters with more abundant warmer-water adapted species.

### Recovered species diversity

**Fig 7:**
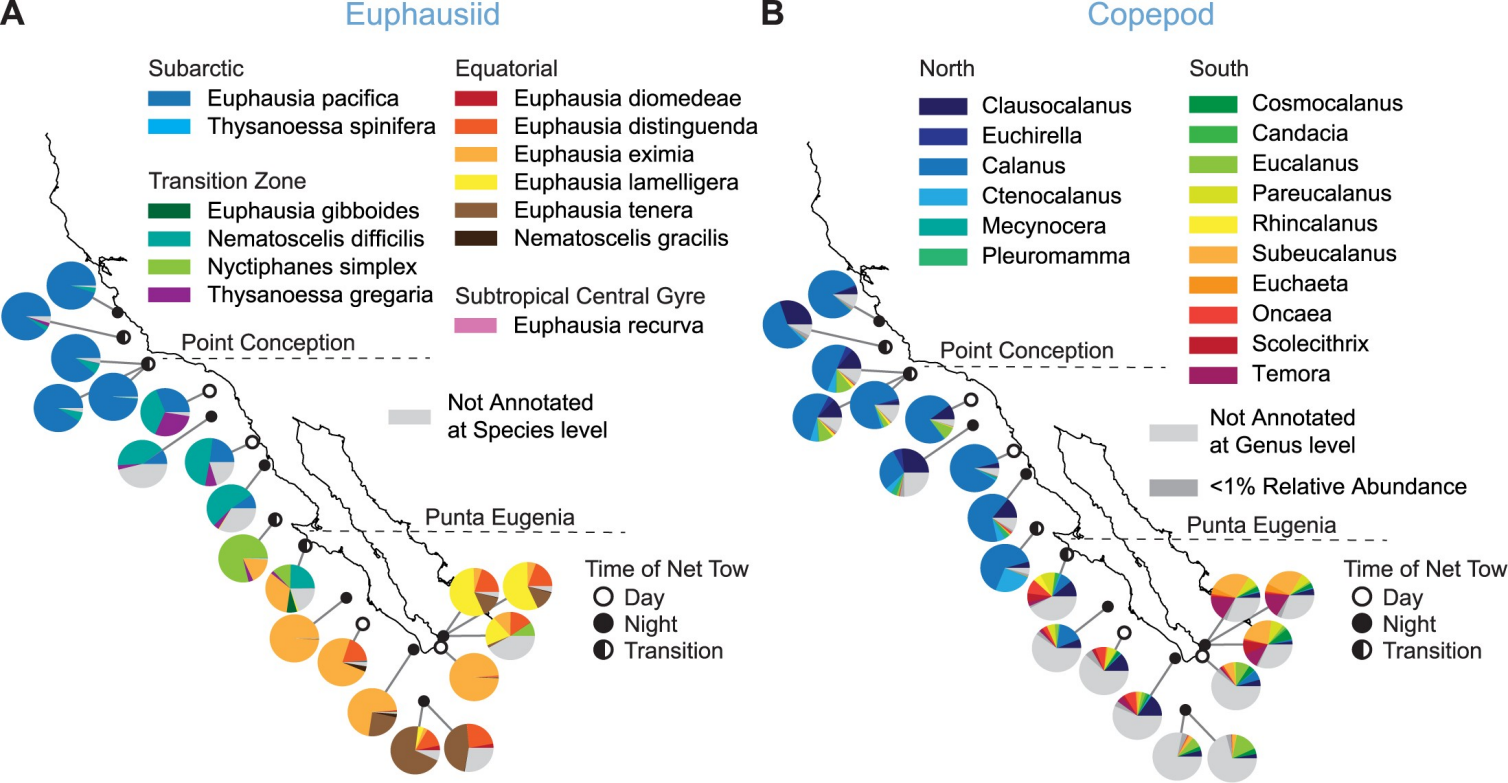
Euphausiid and copepod community composition by sample. (A) Proportion of reads annotated to species out of total reads annotated to the family *Euphausiidae* within each sample from the Banzai COI dataset. Species are organized according to ecological categories from Parés-Escobar, Lavaniegos, & Ambriz-Arreola, 2018. (B) Proportion of reads annotated to copepod genera out of total reads annotated to copepods within each sample from the Banzai COI dataset. Genera are organized in region of highest relative abundance and by Family and Genus. Labeled genera only include genera with ≥ 0.1% total rarefied reads in at least one sample.

Of the diverse zooplankton groups detected, the phylum Arthropoda had the highest relative abundance. Within the COI dataset we recovered a high percentage of local species of copepods and krill (family *Euphausiidae*) allowing us to describe their shifts in community composition (Fig 7). In particular, copepod sequences dominated many of the samples: within the Banzai pipeline results, the highest number of OTUs and the highest number of reads to any species were annotated to the copepod *Calanus pacificus* (COI) or *Calanus* genus (18S). Relative abundance of *C*. *pacificus* was highest in the PCNorth and EUNorth regions where it ranged from more than 64% to a minimum of just over 12% of the *COI* rarefied reads of a sample. At Punta Eugenia, copepod communities transitioned from being dominated by *C*. *pacificus* to a more diverse assemblage (Fig 7B, S3 Fig). Relative abundance patterns of the most common krill species matched those reported for the region, with *Euphausia pacifica* dominating northern samples, *Nematoscelis difficilis* more frequent in northern Baja California to Punta Eugenia, and *Euphausia eximia* dominating south of Punta Eugenia to Cabo San Lucas where Gulf of California waters and tropical surface waters meet (Fig 7A). South of Punta Eugenia we also found the community shifts to include more equatorial species (*Euphausia diomedeae*, *E*. *distinguenda*, *E*. *lamelligera*, *E*. *tenera*, and *Nemotoscelis gracilis*).

We also found evidence of parasitism within the organisms dominating this data set. We recovered parasitic dinoflagellate sequences within the orders Syndiniales and Blastodiniales as well as several families of parasitic copepods: *Clausidiidae*, *Pandaridae*, *Sapphirinidae*, and *Splanchnotrophidae*. Within the dinoflagellates in *Syndiniaceae*, we recovered a genus that parasitizes ciliates (*Duboscquella)* as well as a genus that parasitizes decapod crustaceans (*Hematodinium*). Blastodiniales species typically parasitize copepods. Since these samples were from tows with a 200-μm mesh zooplankton net and based on the small sizes of these dinoflagellates, we assume we recovered mainly organisms present within the bodies of these taxa rather than any free-living or spore stages in the parasitic life cycle, although we cannot exclude the possibility of eDNA or adherent spores. The families of parasitic copepods detected here can generally infect fish and invertebrate species; and were mainly present in EUSouth samples. This region also contained a greater diversity of fish species within our dataset (Fig 6).

Time of day of sampling likely also had an effect on our recovered species diversity however, since this study did not have replication at different times within the same region we are unable to comment on how precisely time of day affected our sampling throughout the study. Within the euphausiids, there is variation in vertical migration as well as net avoidance between California Current species [19]. Despite this source of variation and expected differences due to sampling at different times of day, we found high similarity within regions of samples taken during night and day from the top 100m.

## Discussion

Metabarcoding of mixed tissue samples allowed us to detect a diverse array of marine zooplankton and examine changes in community structure across several different phyla. Our sampling from Monterey Bay to the Gulf of California spanned latitudinal variability in environmental variables such as temperature and salinity, and crossed two documented biogeographic breaks in marine community structure: Point Conception and Punta Eugenia. A clear break in zooplankton community structure occurred at Punta Eugenia but not at Point Conception. Diversity increased with increasing temperature and decreasing latitude across the transect, consistent with global trends [20]. Our major conclusions remained the same across different clustering and taxonomic annotation methods implemented within the Banzai and USEARCH bioinformatic pipelines. Applying genetic primers that amplify a broad range of taxa allowed us to detect many species that traditionally require a large effort to identify and enumerate. As discussed below, application of these methods enabled clear descriptions of major latitudinal trends in zooplankton species diversity and community structure, capturing many of the same species patterns that have been historically documented in the region.

### Community change at Punta Eugenia

The most significant change in overall community structure observed in this study occurred at Punta Eugenia (Figs 3 and 4). These changes probably result from offshore diversion of the California Current at Punta Eugenia, and the intrusion of equatorial, subtropical, and Gulf of California waters. Though we did not recover a strong break in community composition at Point Conception, this result might be a consequence of the time of year of our sampling which was conducted during the spring of 2012. Samples taken at other times might show greater shifts in community composition at Point Conception due to seasonal development of the Southern California Eddy. Nonetheless, despite this temporal snapshot of diversity along this transect, we recovered many well-documented patterns of zooplankton composition (particularly for euphausiids and copepods) observed across years of sampling that included different seasons (discussed below).

Since clustering and diversity analyses were performed on an OTU basis instead of by merging by taxonomic annotation, results take into account intraspecific genetic diversity as well as sequences that were unable to be annotated to the species level. Diversity and community composition can be biased by OTU clustering method [21]. Here we compared results from the program swarm within Banzai [22] with the combination of UNOISE [23] and clustering by sequence similarity within USEARCH. Results from both Banzai and USEARCH pipelines supported a significant break in community structure at Punta Eugenia and an increase in diversity in southern latitudes. Taxonomic annotations revealed similar trends across groups but did contain differences in specificity of annotations (e.g. Banzai in general annotated taxa more specifically than USEARCH and generated an order of magnitude higher number of OTUs, discussed below). Similarly, choice of metabarcoding marker and primer set biases results towards different sets of taxa [24]. Here, results from two different genetic markers, *18S* and *COI*, revealed the same overall trends but with varying sensitivities to the taxa detected and the specificity of taxonomic annotations permitted by the genetic marker for different groups. Both *COI* and *18S* data produced similar clusters of samples (Figs 3 and 4). Overall, in this study *COI* was able to generate more specific taxonomic annotations than *18S* and was more useful for examining differences in species community composition within this dataset. However, there was generally good concordance between markers in broad ratios of abundance of higher taxonomic groups.

### Increased diversity at lower latitudes

Zooplankton diversity was positively correlated with temperature, aligning with global patterns of higher species diversity at lower latitudes [25]. Increased species diversity south of Punta Eugenia was pervasive across several phyla including arthropods, bony fishes, cnidarians and molluscs (Fig 6A). A greater percentage of unique taxa occurred within the EUSouth region than within the EUNorth or PCNorth regions, indicating reduced taxonomic overlap between EUSouth and the EUNorth and PCNorth regions (Fig 6C, S2 Table). Samples taken from different water masses (including the entrance of the Gulf of California) in the EUSouth region showed a much wider range of temperature and salinity values (Fig 1). Broader variability in these environmental factors might be responsible for some of the increased diversity of the EUSouth region. Nevertheless, despite sampling from these different environments, the EUSouth samples were still more similar to each other than to either the PCNorth or EUNorth samples (Figs 3 and 4).

### Comparison to historical sampling of the region

Comparisons with long-term surveys and established datasets revealed that the present molecular and statistical methods recovered a high proportion of copepod and krill species (Fig 7). Latitudinal changes in euphausiid communities along the western coast of California and Baja California are well documented [13,26,27,28]. Our study recovered a high diversity of species from this range and also reflected well-known latitudinal shifts in species composition (Fig 7A). We identified 13 out of 34 species previously recorded for euphausiid communities off of Baja California in a 10-year time series collected during summer months from 1998–2008 [26]. We detected all of the common (present ≥ 5% of the time) subarctic, transition zone, and equatorial species assemblages, only one species known from the subtropical central gyre, and no warm-temperate cosmopolitan species (S1 Table) [26]. Absent from our dataset were three warm-temperate cosmopolitan species that were frequently encountered in the 10-yr time-series: *Stylocheiron affine*; *Nematobrachion flexipes*; and *Stylocheiron longicorne* (S1 Table). Three additional species of these 34 were assigned to OTUs by lowest common ancestor algorithm but did not meet the sequence identity threshold to be assigned at species level. The absence of these warm-temperate species could be due to conditions at time of sampling in February of 2012.

We also recovered a large proportion of copepod genera known to occur in the sampled region. The composition of copepod communities is commonly interpreted as an indicator of changing oceanographic conditions, and herein their distributions correspond with changing water masses (Fig 1, Fig 7B) [12,29]. CalCOFI (California Cooperative Oceanic Fisheries Investigations) surveys conducted from 1951 to 2015 enumerated 20 copepod genera present in the Central Californian and Southern Californian regions. Our Banzai dataset identified 16 of the 20 genera, and three additional genera had OTUs assigned to them by lowest common ancestor algorithm but with matches of less than 95% sequence identity. The remaining missing genus from our dataset, *Gaussia*, had the lowest abundance within the CalCOFI dataset and was last detected in 2001. We could not determine whether the copepod or euphausiid genera missing from our dataset resulted from absence of these organisms from the region or sample, or were due to the sensitivities of our methods. Although the present “absence of evidence is not evidence for absence,” [30] it is encouraging that the majority of copepod and krill species routinely monitored in this region were recovered with the present methods.

In addition to recovering the distributions of commonly recorded organisms from this region, we also detected with varying levels of specificity cryptic organisms such as parasitic dinoflagellates and copepods known to infect fish and invertebrates. Parasite detection with these molecular methods might be useful for future studies aimed at elaborating their roles in regulating zooplankton communities [31]. Also, detection of species-specific parasites might serve as indicators of the presence of their host species.

In summary, metabarcoding with both *18S* and *COI* markers allowed us to detect: (*i*) community changes at the species level; (*ii*) a significant break in community structure at Punta Eugenia; and (*iii*) cryptic diversity of otherwise undetectable groups. As demonstrated by the detection of DNA from a Blue Whale, the sequences presented here likely derived not only from the tissues of whole organisms caught in our nets, but also from environmental DNA (eDNA), i.e. material left behind by organisms present in the water column. Regardless of this ambiguity, our purpose was to recover patterns of biodiversity and community change across the sampled region. Consequently, eDNA detections also contribute to our knowledge of regional species diversity. Altogether the present results illustrate the utility of metabarcoding to recover patterns of zooplankton and parasite community composition in marine environments.

Across the western coast of North America, the northern and southern range boundaries of marine species can vary interannually. Changes in physical forcing, environmental conditions, and food web interactions cause temperate and tropical species to expand toward the poles during warm years and arctic species to expand toward the equator during cool years [14,16,32,33]. Overall, the year 2012 was characterized by cooler water temperatures within the California Current system [34]. A longstanding time-series of copepod diversity and richness from Newport, Oregon revealed decreased richness and a positive northern copepod biomass anomaly occurred in 2012, consistent with the effect of cooler temperatures [34]. We also found evidence for cooler temperatures influencing zooplankton communities. For example, abundance of the coastal warm-water species *Nyctiphanes simplex* is significantly correlated with decadal environmental patterns within the California Current [13]. Typically its range extends to north of Point Conception, but during el Niño years it can be found as far north as Washington and British Columbia waters [13,35,36]. However, in this study the northern extent of *N*. *simplex* was from two stations at Punta Eugenia, central Baja California (Fig 7A). The dominating northern cold-water species, *Euphausia pacifica*, was a major component of the community until site UC7, just north of Punta Eugenia. This species typically peaks during La Niña years and is much less abundant during warmer el Niño years [13]. Therefore, our results for euphausiids appear to reflect cooler conditions. Future research would greatly benefit from conducting metabarcoding alongside traditional sampling to better calibrate frequency and variability in species distributions. The California Current System not only includes significant interannual variability in biological and physical characteristics, but also has had dramatic climactic changes. After a shift to a warmer northeast Pacific Ocean after 1976, the Southern Californian region has experienced a dramatic increase in temperature [37]. Improving our ability to describe biodiversity across many different groups of organisms will allow a better understanding of how these communities vary under changing long-term environmental conditions.

### Benefits and limits of metabarcoding of environmental samples

Metabarcoding methods hold great promise to enable relatively quick and inexpensive identification of previously unrecoverable biological diversity. These methods rely on the accuracy of genetic reference databases, and are affected by methodological factors, ranging from biases in primer amplification to bioinformatic choices for processing of sequence data, that influence the enumeration of species diversity [38]. Metabarcoding methods are also unable to recover information about life stage or health of organisms (although as shown by this study they can reveal the presence of parasitic organisms). Considerably more research must be conducted to relate sequence abundance to biomass. For example, metabarcoding methods can be biased by variation in gene copy number, artificially inflating the relative abundance of some taxa over others, and a gravid female would be overrepresented in a genetic dataset compared to a morphological one. However, sample bias also exists with traditional enumeration methods, resulting from sampling methodology and the taxonomic expertise and breadth of scientists engaged in identifications [39]. It was encouraging that within this dataset our conclusions were consistent for both *18S* and *COI* markers and between two different bioinformatic pipelines. The utility of metabarcoding is evident in its ability to recover species diversity across many broad taxonomic groupings, to identify shifts in species composition previously recorded in the scientific literature, and to identify the presence of cryptic organisms that would otherwise be missed with traditional methods.

## Conclusion

Metabarcoding analysis of mixed tissue samples obtained with net-tows along the California and Baja California margins identified a clear shift in community composition at Punta Eugenia that was absent at Point Conception. Species diversity increased with decreasing latitude and correspondingly increasing temperatures. These conclusions were stable across both *18S* and *COI* genetic markers and two bioinformatic pipelines. We also recovered well-documented latitudinal shifts in euphausiid and copepod species as well as ecologically informative groups such as parasites that might otherwise have been missed by traditional sampling. The ability of metabarcoding to detect diverse zooplankton groups illustrates its utility in detecting changes in zooplankton diversity over environmental gradients. Shifting zooplankton community structure can be used as a primary indicator of changes in climatological or physical patterns as well as having broad implications for food web structure and health of higher trophic level organisms. Since metabarcoding analyses allow detection of many more species than traditional means across a broad taxonomic range, we show it can be a valuable method for detecting geographic variation in populations.

## Materials and methods

### Sample acquisition

An R/V *Western Flyer* expedition (Monterey Bay Aquarium Research Institute, Moss Landing, CA, USA) obtained zooplankton samples from 15 stations between Moss Landing, CA, and La Paz, Mexico from February 4^th^ to 12^th^ of 2012 (Fig 1, Table 1). Field collections were taken under permit number CTC-001340 granted by the government of Mexico. We towed a 75cm-diameter 200-μm mesh zooplankton net obliquely from a depth of 100 meters to the surface. Net tows were taken soon after arrival at each station. Consequently, we classified the samples as night (after sunset), day (after sunrise), or sunrise/sunset (within 2 hours of sunrise or sunset) (Table 1). The cod-end samples were filtered immediately through a 100 μm sieve and preserved in 95% EtOH. We used PowerSoil® DNA Isolation Kits (Qiagen, Germantown, MD) to isolate total DNA from approximately 200 mg subsamples from each station. NanoDrop 1000 spectrophotometer (ThermoFisher Scientific, Waltham, MA) measurements were used to normalize DNA extracts to a final concentration of 20 ng/ul. Replicate DNA extractions were conducted at three stations (UC3, GOC2, and CP23) (3, 2, and 3 samples respectively). These replicates were carried through all analyses. Although there were some differences in relative abundance of taxonomic groups (Fig 2), in clustering analysis variation between replicates was smaller than between samples (Figs 3 and 4).

### Library preparation

Genomic DNA was quantified with the Invitrogen Quant-iT^TM^ PicoGreen^TM^ dsDNA Assay Kit (Thermo Fisher Scientific, Cat. No. P7589) to determine the double-stranded DNA concentration following the manufacture’s protocol and then standardized to 2.5 ng μL^-1^ for a two-step PCR amplification. The mitochondrial cytochrome c oxidase subunit I (*COI*) gene of each plankton sample was amplified, in triplicate, using primers mlCOIintF forward (5’- GGWACWGGWTGAACWGTWTAYCCYCC -3’) [40] and jgHCO2198 reverse (5’- TAIACYTCIGGRTGICCRAARAAYCA-3’) [41]. The small subunit ribosomal RNA (*18S* rRNA) gene was also amplified in triplicate using primers SSUF04 forward (5’-GCTTGTCTCAAAGATTAAGCC-3’) and SSUR22 reverse (5’–GCCTGCTGCCTTCCTTGGA-3’) [42]. Both primer sets had partial Nextera barcode indices added to the 5’ ends (Illumina support 2013).

In the primary PCR reaction, 2.5 ng of genomic DNA from each sample was amplified with final concentration of 1X Kapa Robust Hot Start Ready Mix (KAPA Biosystems, Cat. No. 07961383001), 0.2 mg mL^-1^ BSA, 2 mM MgCl_2_, and 0.4 μM of each primer in a 25-μL reaction. Thermocycling conditions consisted of an initial 3-minute cycle at 95°C, followed by 27 cycles of 1 minute at 95°C, 45 seconds at 47°C, and 1 minute at 72°C with a final 72°C hold for 5 min. Amplicons were viewed on a 1.5% agarose gel stained with ethidium bromide under UV light. Triplicates were pooled and purified with 1.4 times of the PCR product volume with Agencourt AMPure XP beads (Beckman Coulter Life Sciences, Part No. A63881) according to the manufacturer’s protocol.

The second-step PCR amplification involved attaching the Nextera barcodes on the primary PCR product for Illumina sequencing. DNA concentration of the pooled and purified first-round PCR product was quantified with the Invitrogen Quant-iT^TM^ PicoGreen^TM^ dsDNA Assay Kit and then standardized to 2.5 ng μL^-1^. One microliter of the pooled, purified, and standardized amplicons was amplified in a PCR cocktail comprising a final concentration of 1X Kapa Robust Hot Start Ready Mix, 0.2 mg mL^-1^ BSA, 0.2 μM each forward and reverse barcode, and 2 mM MgCl_2_ in a final volume of 25 μL. Thermocycling conditions consisted of an initial 3-minute cycle at 95°C, followed by 8 cycles of 30 seconds at 95°C, 30 seconds at 55°C, and 30 seconds at 72°C with a final 72°C hold for 5 minutes. PCR products were viewed and purified as in the primary PCR reaction.

DNA concentration of the barcoded and purified *COI* and *18S* amplicons were separately quantified with the Invitrogen Quant-iT^TM^ PicoGreen^TM^ dsDNA Assay Kit, standardized to the lowest DNA nanomolar (nM) concentration, and 10 μL of each standardized sample pooled into a sterile 1.5 mL microcentrifuge tube. The pooled library was purified again with 1.4 times of the pooled library volume with the Agencourt Ampure^TM^ beads, according to the manufacturer’s protocol, and eluted in low Tris-EDTA buffer. One microliter of the pooled library was loaded on a High Sensitivity DNA chip (Aligent Technologies, Cat. No. 5067–4626) and quantified with the Agilent 2100 Bioanalyzer System to determine the final concentration. A qPCR assay was also performed as a secondary library quantification method using the KAPA Library Quant Kit^TM^ (KAPA Biosystems, Cat. No. KK4835) according to the manufacturer’s protocol.

### Illumina sequencing

The *COI* and *18S* combined libraries were diluted to 4 nM and sequenced on an Illumina *MiSeq* system using an Illumina *MiSeq* v3 kit (600 cycles, Illumina, Cat. No. MS-102-3003) and an aliquot of a PhiX Control v3 reagent (Illumina, Cat. No. FC-110-3001) in the GTAC Lab at San Francisco State University in May 2017. The library was denatured with 0.2 normality (N) sodium hydroxide, combined with 20% PhiX (also denatured with 0.2 N sodium hydroxide), and diluted to a final concentration of 8 pM before loading it in the cartridge according to the manufacturer’s recommended protocols.

### Bioinformatic analyses

Two bioinformatic pipelines of analysis were used to analyze the resulting Illumina sequencing data: the first adapted from the *banzai* pipeline [43] and the second based on the USEARCH pipeline [44]. Principle differences include varying methods of OTU clustering (swarm at d = 1 or UNOISE to 99% then clustering at 95%/97% similarity (*COI/18S*)) and taxonomy assignment (based on lowest common ancestor (LCA) algorithm in MEGAN6 using blast hits to GenBank or top blast hit to a custom database/GenBank (*COI/18S*)).

#### Banzai pipeline

The first bioinformatic pipeline was adapted from the *banzai* pipeline that links together bioinformatic programs through a shell script [43]. Complete script and parameters are included in supplemental methods. Reads were merged through PEAR [45], quality filtered through VSEARCH [46], demultiplexed using awk, primers removed through cutadapt [47], dereplicated, and clustered using swarm with d = 1 [22]. Chimeras were removed using VSEARCH. Taxonomy was assigned through blastn searches to NCBI GenBank’s non-redundant nucleotide database (nt). Blast results were filtered using MEGAN6’s lowest common ancestor (LCA) algorithm [48]. Only hits with ≥80% sequence identity, ≥200 bitscore for *COI* (≥300 bitscore 18S) and whose bitscores were within the top 2% of the highest bitscore value for each OTU were considered by MEGAN6. The MEGAN6 parameter LCA percent was set to 0.8, allowing for up to 20% of top hits to be off target and still have the majority taxonomy assigned. This parameter value was chosen to allow for minor numbers of incorrectly annotated GenBank entries–effectively allowing for OTUs which had many high-quality hits to a taxa to still be assigned to that taxa even if there existed a high-bitscore hit to another GenBank sequence annotated to an unrelated taxa. We decided this was more advantageous than the disadvantage caused by ignoring small numbers of true closely related sequences. Furthermore, post-MEGAN6 filtering was performed to ensure only contigs with a hit of ≥97% sequence identity and ≥400 bitscore for *COI* (≥600 bitscore *18S*) were annotated to the species level. Only contigs with a hit of ≥95% sequence identity and ≥300 bitscore for *COI* (≥550 bitscore *18S*) were annotated to the genus level. Annotations were elevated to the next highest taxonomic level for contigs that failed those conditions. Contigs were size limited to exclude those that were <250nt or ≥350nt for *COI* and <340nt and ≥415nt for *18S*. Contigs annotated to human (Genus *Homo*), pig (Family *Suidae*), insect (Class *Insecta*), spider (Class *Arachnida*), Bacterial, or Archaea groups were removed. For each OTU that was present within the negative control, the number of reads in the control was subtracted from all environmental samples. Samples were rarefied within the program phyloseq in R to the lowest read number present within the environmental sample set (*COI*: 129,363; 18S: 28,676)[49].

#### USEARCH pipeline

The second bioinformatic pipeline used USEARCH v10.0 [44]. Paired-end reads were merged (-fastq_mergepairs) using a minimum final length of 356 bp, a maximum final length of 374bp, and 12 differences allowed in the merge alignment. These parameters were chosen to allow for variation in the size of the *COI* fragment, in the case of the maximum and minimum final lengths, and to follow the software recommendations for read pairs with long, overlapping merge areas (>100bp). To filter low quality reads from the dataset, we used a strict maximum expected error rate of 0.5 (-fastq_filter). Primers were trimmed from the ends of all reads (-fastx_truncate). Sequences were then dereplicated (-fastq_uniques) and sorted by abundance (-sortbysize). Singletons were removed at this step to increase the speed of clustering. Clustering was conducted in two steps. First, sequences were clustered at 99% similarity (zotus) using UNOISE [23], which also includes chimera detection and removal. Sequences were then sorted by length (-sortbylength) and clustered a second time at 95% similarity (-cluster_smallmem) to approximate metazoan species richness with genetic similarity across the *COI* gene region. The *18S* amplicon sequencing data were processed through most of the same USEARCH pipeline except that 1) the minimum and maximum final sequence lengths programmed for merging the paired end reads were 250bp and 450bp, respectively; and 2) the zotu reads were clustered at 97% similarity.

The resulting OTU tables for the *COI* and *18S* genes were rarefied to 166417 and 85279 reads per sample respectively, using the Plymouth Routines in Multivariate Ecological Research (PRIMER) v7 software [50]. The GOC2b replicate sample was excluded within the *18S* dataset since it would have lowered the number of rarefied reads per sample to 51878. In addition, the representative OTU sequences from each pipeline had been assigned taxonomy based on a modified *COI* genetic reference database and an *18S* genetic reference database from GenBank.

#### Pipeline comparison

All contigs from unrarefied datasets from both pipelines and markers were used in reciprocal blastn searches to compare genetic diversity and taxonomic assignments of the resulting datasets. Taxonomic annotations and regional analyses of species composition patterns were also compared across both pipelines.

### Regional comparison and multivariate analysis

Zooplankton samples were categorized into three regions based on their geographic locations with respect to the two historical biogeographic boundaries: “PCNorth” (North of Point Conception), “EUNorth” (North of Punta Eugenia), and “EUSouth” (south of Punta Eugenia) (Fig 1, Table 1). Results of the multivariate and diversity analyses are based on OTUs and presented by gene. Effective diversity metrics were calculated using the vegan package in R as the exponent of Shannon diversity [51,52]. A linear model was fitted in R and using the package segmented break point analyses was conducted but no significant breaks were found (Banzai COI; USEARCH COI) or convergence was not attained (Banzai 18S, USEARCH 18S). Therefore a single linear model was determined to be the best fit. Multivariate analyses were conducted using the packages phyloseq, vegan and clustsig in R [49,51,53,54]. Data were manipulated and compositional figures were generated using the packages pandas, numpy, and matplotlib in python [55–58]. Circular phylogenetic trees and figures were plotted using GraPhlAn [59]. Figure colors and fonts were edited and composite figures created in Adobe Illustrator.

## Supporting information

S1 Fig Detection of genus-level annotations across both markers and pipelines. Detection of genus-level annotations across both 18S and COI marker sets and across both pipelines (Banzai (B) and USEARCH (U)). Colored bar indicates genus was detected within that dataset.
(TIF)

S2 Fig Correlation between temperature and effective diversity (18S). Banzai 18S data. Mean diversity value of replicates was taken to represent sites UC3, CP23, and GOC2.
(PNG)

S3 Fig Correlation between temperature and effective diversity of copepods (COI). Banzai COI data limited to Class Hexanauplia. Mean diversity value of replicates was taken to represent sites UC3, CP23, and GOC2.
(PNG)

S1 Table Krill species detected by marker. Krill species detected within 18S and COI Banzai datasets, their abundance in the datasets, and whether they are predicted to be present in the region.
(XLSX)

S2 Table Percent unique taxa by OTU and by annotation. Percent unique taxa calculated by shared presence/absence of OTU sequences or by presence/absence of taxonomic annotations for both 18S and COI Banzai datasets. Data calculated for individual samples, sites, and by region.
(XLSX)
