# Supplementary figures and images for: Zooplankton biogeographic boundaries in the California Current System as determined from metabarcoding
Source: PLoS One. 2020 Jun 25;15(6):e0235159. doi: 10.1371/journal.pone.0235159 (PMC7316296; doi:10.1371/journal.pone.0235159)

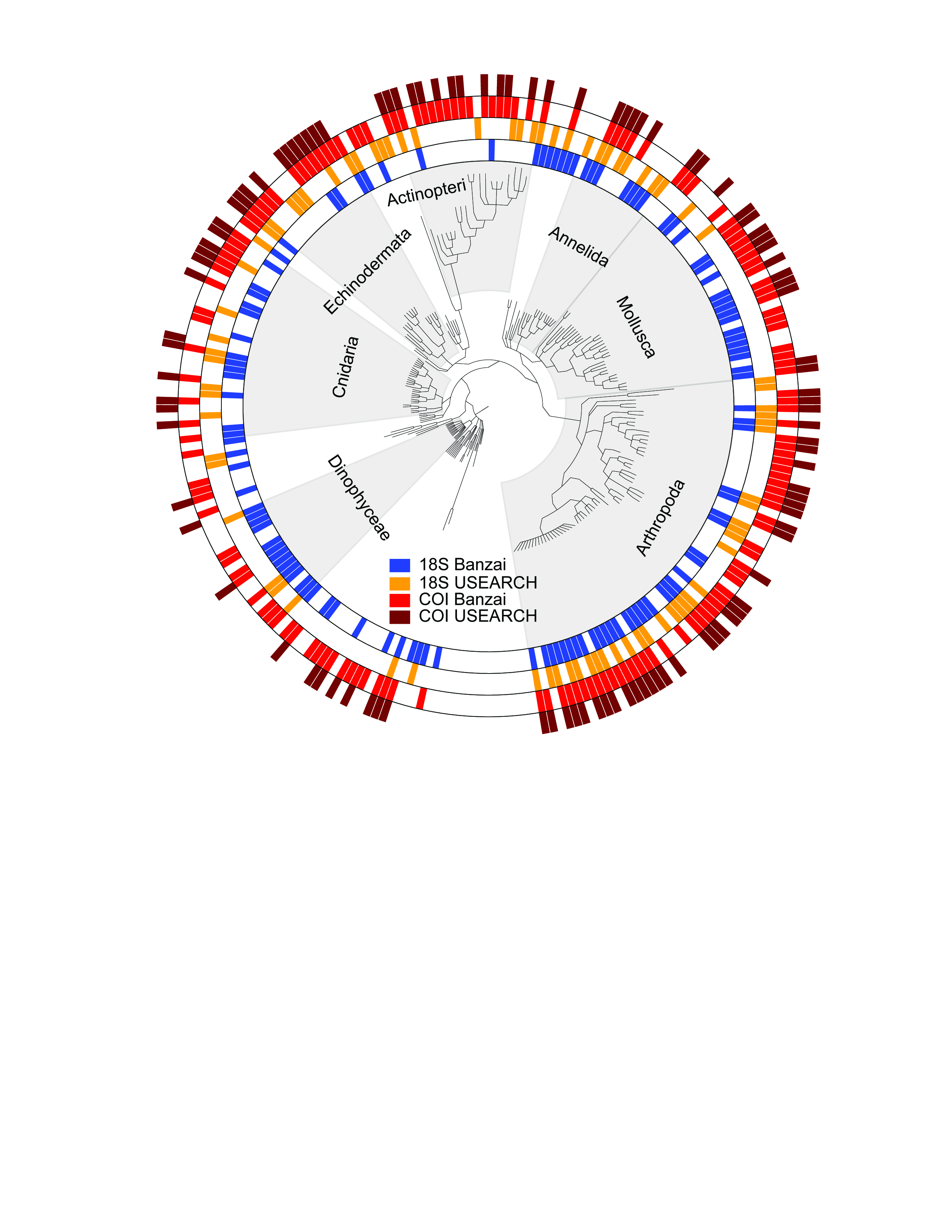

Supplement: S1 Fig — Detection of genus-level annotations across both 18S and COI marker sets and across both pipelines (Banzai (B) and USEARCH (U)). Colored bar indicates genus was detected within that dataset. (TIF) [file pone.0235159.s001.tif]

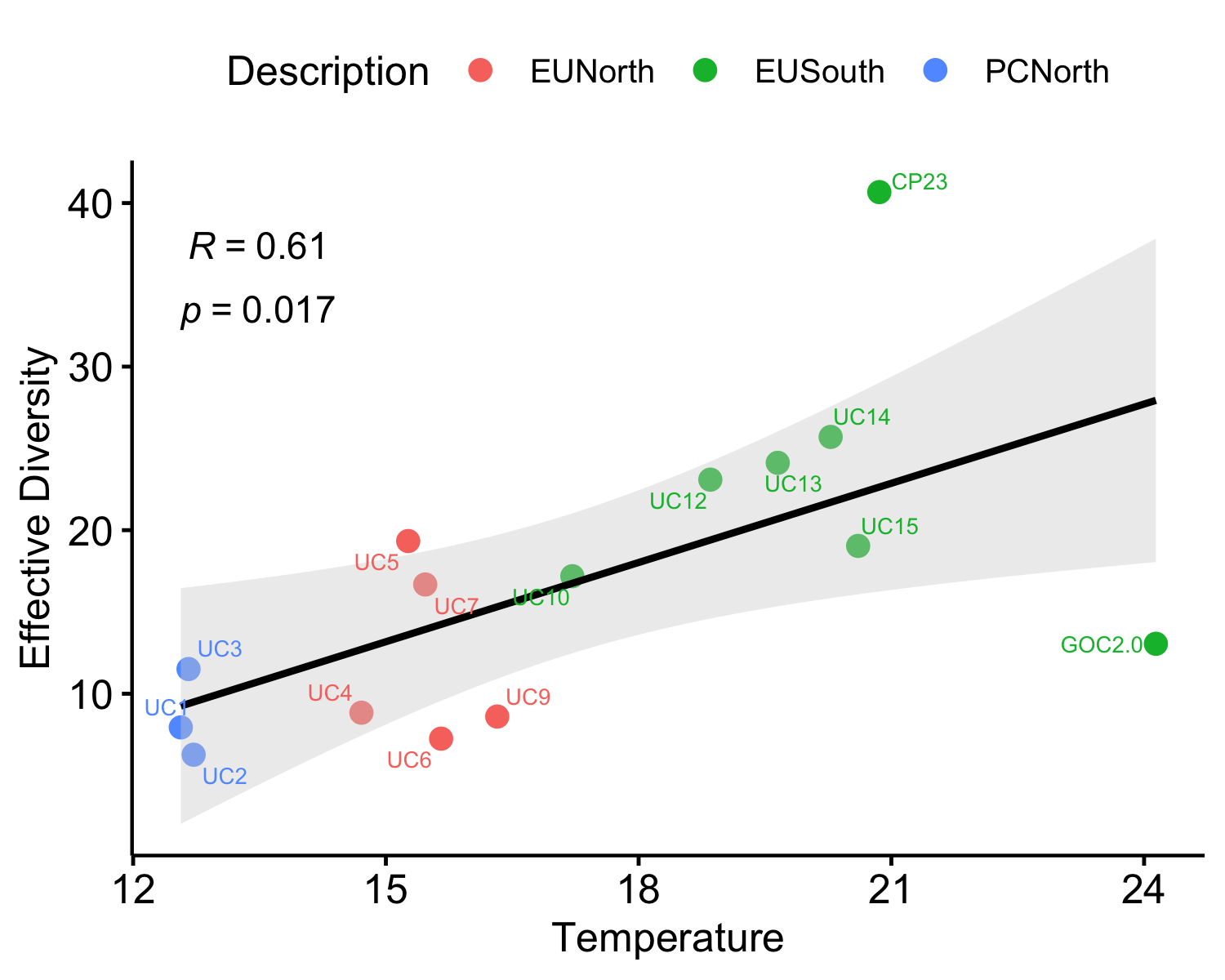

Supplement: S2 Fig — Banzai 18S data. Mean diversity value of replicates was taken to represent sites UC3, CP23, and GOC2. (PNG) [file pone.0235159.s002.png]

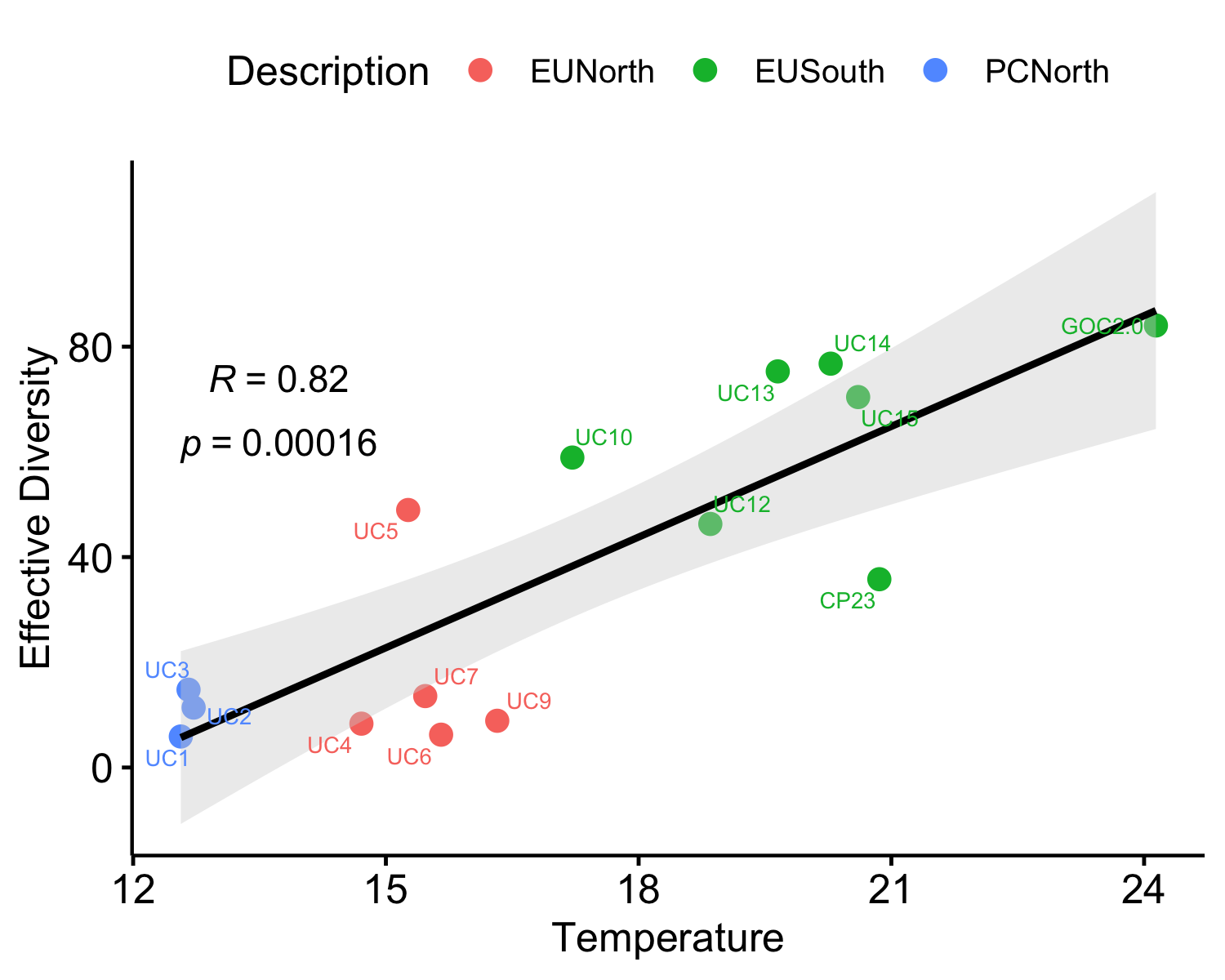

Supplement: S3 Fig — Banzai COI data limited to Class Hexanauplia. Mean diversity value of replicates was taken to represent sites UC3, CP23, and GOC2. (PNG) [file pone.0235159.s003.png]
